# A Highly Sensitive Diagnostic System for Detecting Dengue Viruses Using the Interaction between a Sulfated Sugar Chain and a Virion

**DOI:** 10.1371/journal.pone.0123981

**Published:** 2015-05-26

**Authors:** Budi Saksono, Beti Ernawati Dewi, Leonardo Nainggolan, Yasuo Suda

**Affiliations:** 1 Graduate School of Science and Engineering, Kagoshima University, 1-21-40, Kohrimoto, Kagoshima, 890–0065, Japan; 2 Indonesian Institute of Sciences, Research Center for Biotechnology, Jl. Raya Bogor, Km. 46, Cibinong, 16911, Indonesia; 3 Department of Microbiology, Faculty of Medicine, Indonesia University, Jl. Pegangsaan Timur No. 16, Jakarta, 10320, Indonesia; 4 Department of Internal Medicine, Faculty of Medicine, RSUPN Cipto Mangukusumo, Jl. Diponegoro No. 71, Salemba, Jakarta Pusat, Indonesia; Centro de Pesquisas René Rachou, BRAZIL

## Abstract

We propose a novel method of detecting trace amounts of dengue virus (DENVs) from serum. Our method is based on the interaction between a sulfated sugar chain and a DENV surface glycoprotein. After capturing DENV with the sulfated sugar chain-immobilized gold nanoparticles (SGNPs), the resulting complex is precipitated and viral RNA content is measured using the reverse-transcription quantitative polymerase chain reaction SYBR Green I (RT-qPCR-Syb) method. Sugar chains that bind to DENVs were identified using the array-type sugar chain immobilized chip (Sugar Chip) and surface plasmon resonance (SPR) imaging. Heparin and low-molecular-weight dextran sulfate were identified as binding partners, and immobilized on gold nanoparticles to prepare 3 types of SGNPs. The capacity of these SGNPs to capture and concentrate trace amounts of DENVs was evaluated *in vitro*. The SGNP with greatest sensitivity was tested using clinical samples in Indonesia in 2013–2014. As a result, the novel method was able to detect low concentrations of DENVs using only 6 μL of serum, with similar sensitivity to that of a Qiagen RNA extraction kit using 140 μL of serum. In addition, this method allows for multiplex-like identification of serotypes of DENVs. This feature is important for good healthcare management of DENV infection in order to safely diagnose the dangerous, highly contagious disease quickly, with high sensitivity.

## Introduction

Dengue viruses (DENVs), which have 4 phylogenetically and antigenically distinct types (DENV-1 to DENV-4), are emerging arthropod-borne flaviviruses that cause dengue fever (DF), dengue hemorrhagic fever (DHF), or dengue shock syndrome (DSS), mostly in tropical and subtropical countries [1]. More than 200 million cases of DF are registered annually, and 1–5% of these progress to more severe forms. In 2009, the World Health Organization classified DENV-infected patients as having “dengue without warning signs,” “dengue with warning signs,” or “severe dengue” [2]. A large body of data suggests that severe dengue often, but not always, occurs during secondary heterotypic DENV infections in adults and children via antibody-dependent enhancement (ADE) [3–6]. All DENV subtypes are distributed throughout the world, including Indonesia, and co-circulation of multiple DENV types has been reported [7, 8]. Co-occurrence of multiple types may therefore lead to an increase in the number of severe dengue cases. Even without infection by multiple virus types, severe dengue often occurs from primary infections in infants. Unfortunately, no approved vaccines or effective antiviral drugs against DENV are available. Therefore, a highly sensitive, accurate, and convenient diagnostic test for a DENV infection should be performed in order to initiate treatment and specific preventive measures.

Current DENV diagnostic methods are based on serological detection of circulating antibodies. Specificity of these tests suffer due to cross-reactivity from antibodies against related flaviviruses, thus they are insufficient. Alternative methods for the detection of DENV rely on the measurement of viral RNA using reverse transcription polymerase chain reaction (RT-PCR). RT-PCR [9–12] has some advantages, such as high sensitivity and specificity, and the ability to differentiate serotypes. There are some disadvantages, including the need for trained staff and complex equipment, and the increased sensitivity leading to an increased risk of false-positives due to contaminated samples. With respect to early diagnosis of DENVs, research efforts have been directed toward technological improvements such as utilization of a charged polymer for trapping the virus [13], PCR in combination with whole blood spotted on filter paper [14], and serotyping the DENV [15]. No improvements in concentrating DENV for increased sensitivity of DENV detection have been reported yet.

Our innovative approach is based on exploiting the interaction of a DENV envelope protein (glp E) with sulfated sugar chains. It was reported that dengue virions comprise 180 identical copies of envelope proteins, which cover the viral surface [16, 17]. Infection is initiated when the viral envelope proteins attach to a cell surface [18]. The envelope proteins belong to the glycoprotein family, and many reports have described the interaction as taking place between the viral proteins and sulfated polysaccharides, such as heparan sulfate [19–21], fucoidan [22], and sulfated α-d-glucan [23].

Here, we describe a novel method to capture and concentrate DENVs by sugar chain-immobilized gold nanoparticles (SGNP), followed by PCR-based detection. First, we identified DENV-binding sugar chains to be used for capture and concentration of DENV. Second, the selected sugar chains were immobilized on gold nanoparticles to prepare SGNPs [24, 25]. Third, the capacity of SGNPs to capture and concentrate DENVs was evaluated *in vitro*. Finally, we compared our novel SGNP method with a commercially available RNA extraction kit to detect DENV in patient serum and plasma samples in a clinical setting using reverse-transcription quantitative PCR (RT-qPCR).

## Materials and Methods

### Ethics Statement

Ethical approval (63/PT02.FK/ETIK/2010) was provided by the Ministry of Health of the Indonesian Government, and written informed consent was obtained from all human subjects. All samples were a part of the Community Base Dengue Study (CBDS) conducted by the Indonesia University Cipto Mangun Kusumo Hospital. The samples were stored at −70°C until analysis.

### Viruses

DENVs (Types 1, 2, and 4) were kindly provided by Drs. Kase and Hiroi at Osaka Prefectural Institute for Public Health. DENVs were cultivated in freshly cultured Vero cells.

### SPR Imaging

A freshly prepared and purified DENV solution from the culture supernatant of DENV-infected Vero cells was diluted (1:20) with PBS-T. These diluted DENV samples were run through the SPR device at a flow rate of 150 μL/min. After 60 s, the buffer was changed to PBS-T with the same flow rate for 3 min to analyze dissociation. Binding was monitored using the CCD camera in the SPR imaging system (MultiSPRinter, Toyobo, Osaka, Japan). After each run of 1 sample, the chip was washed with 10 mM NaOH for 2 min and re-equilibrated with PBS-T for 5 min.

### Preparation of SGNPs

Based on the results from SPR imaging, and due to their common availability, heparin and low-molecular-weight dextran sulfate were selected and immobilized on gold nanoparticles to prepare Hep-cGNP and DS25-cGNP, respectively. Two conjugation methods were used, as previously reported [15, 16, 20, 21], with slight modifications. In brief, an aqueous solution of 16.2 mM sodium citrate was added to aqueous 1.1 mM NaAuCl_4_, with stirring, at 100°C. The heparin-linker conjugate or the dextran sulfate-linker conjugate was then added to the solution with gentle stirring, and the solution was stirred for 1 h at 25°C in the dark. The solution was centrifuged at 12,000 × *g* for 40 min to obtain a precipitate. After several washes with double-distilled water (ddH_2_O), the precipitate was re-suspended in ddH_2_O and adjusted to an optical density (A_530_) of 0.5 using a cuvette with a 1-mm long light path, to obtain Hep-cGNP or DS25-cGNP.

As an alternative to produce low-molecular-weight dextran sulfate-immobilized gold nanoparticles, 50 mM of sodium tetrahydroborate was added to 1.3 mM NaAuCl_4_ with stirring at 4°C. The linker-dextran sulfate conjugate was then added to the solution, and incubated with gentle stirring for 1 h at 4°C in the dark. The solution was dialyzed against ddH_2_O using a dialysis membrane with MW 3500 cutoff and then lyophilized. The lyophilized powder was re-suspended in ddH_2_O and adjusted to the concentration of 10 mg/mL. Sequential centrifugation was performed at 12,000 × *g* for 15 min to obtain the precipitate. The precipitate was re-suspended in ddH_2_O and adjusted to 1 mg/mL with ddH_2_O to obtain the DS25-GNP solution.

The concentrations of heparin and low-molecular-weight dextran sulfate were measured using the p-aminobenzoic acid ethyl ester method [5]. Heparin content was 2% in Hep-cGNP, and the proportion of low-molecular-weight dextran sulfate was 1% in DS25-cGNP, and 9% in DS25-GNP. The size of the nanoparticles in ddH_2_O was measured by dynamic light scattering (Zeta-Sizer Nano-ZS, Melbane, Kobe, Japan). On average, Hep-cGNP particles had a diameter of 60 nm, DS25-cGNP a diameter of 48 nm, and DS25-GNP a diameter of 27 nm.

### Capture and concentration of DENV, and the release of DENV RNA *in vitro*

Five hundred micro-liters of the DENV-1 culture supernatant was diluted 1:10^4^ or 1:10^6^ with PBS and mixed with 10 μL of SGNP. This mixture was incubated at room temperature with shaking for 30 min. The mixture was then centrifuged (10,000 ×*g* for 10 min at 4°C) to separate the precipitate. The precipitate was washed with PBS and treated with 20 μL of RNase-free water containing 0.1% SDS to disrupt virus particles, and then centrifuged again at 10,000 ×*g* for 10 min at 4°C to remove SGNPs.

### Capture of DENV from patient serum or plasma samples, and release of DENV RNA

Six micro-liters of a patient’s serum or plasma was mixed with 600 μL of PBS at room temperature. Five hundred micro-liters of the above solution was then mixed with 10 μL of DS25-GNP and incubated at room temperature with shaking for 30 min. The mixture was then centrifuged (10,000 ×*g* for 10 min at 4°C) to separate the precipitate from the supernatant. The precipitate was washed with PBS and treated with 20 μL of RNase-free water containing 0.1% SDS to disrupt viral particles. The mixture was centrifuged again at 10,000 × *g* for 10 min at 4°C to remove SGNPs.

### Extraction of Qiagen kit-eluted RNA

RNA was extracted from 140 μL of serum/plasma samples or viral culture supernatants using a QIAamp Viral RNA Mini Kit (Qiagen) according to the manufacturer’s instructions. RNA was eluted in 40 μL of the supplied elution buffer.

### Real-time PCR Amplification

Two micro-liters of eluate from the Qiagen kit elution, as well as the precipitate-derived RNA solution, the separated supernatant, and the original solution without SGNP were used as templates for real-time PCR amplification. The primers were prepared according to Poovoraman *et al* [12] (S2 Table). RT-qPCR was performed using the One-Step SYBR Prime Script RT-PCR Kit 2 (Perfect Real Time, Takara Bio Inc., Otsu, Japan), with Tween 20 (final concentration 0.05%) added to the reaction mixture. The mixtures were incubated for 5 min at 45°C for RT, followed by 40 PCR cycles of 5 sec at 95°C and 30 sec at 60°C, using either the Thermal Cycler Dice Real-Time System (Takara Bio Inc.) or iQ5 Real Time PCR Detection System (Bio-Rad Laboratories, Hercules, CA, USA).

### SD Bioline dengue Duo NS1 and IgG/IgM tests

All tests in this study were carried out in accordance with the manufacturer’s instructions. Briefly, for the SD Bioline NS1 Ag test (Standard Diagnostics, Inc., Yongin, Republic of Korea), 100 μL of serum was added into the sample well of the device. The test results were recorded after 15–20 min. Similarly, in the SD Bioline IgG/IgM test, 10 μL of serum was added into the sample well of the device. We then added 4 drops (~120 μL) of the assay diluent to the round-shaped assay diluent well. The results were recorded after 15–20 min. The test results were analyzed and interpreted according to the manufacturer’s instructions.

## Results

### Screening of sugar chains that specifically bind to DENVs

**Fig 1:**
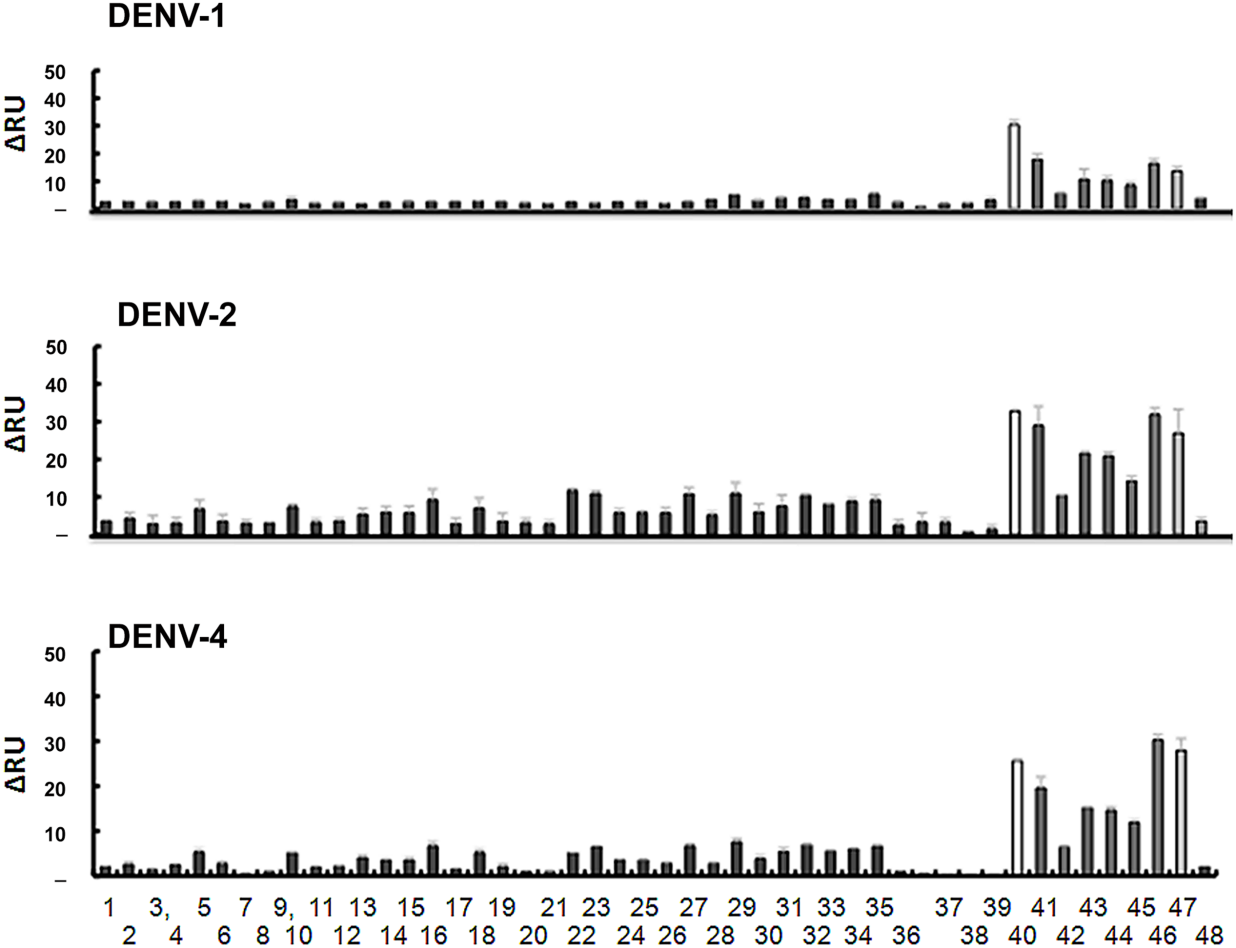
Binding affinities of dengue viruses (DENVs) for various sugar chains immobilized on an array-type Sugar Chip. The map of immobilization, an example of the data on the binding, and the list of sugar chains are shown in S1 Fig. DENVs re-suspended in PBS-T at the concentration of 1.5–12 μg/mL (total viral protein) were loaded onto the sugar chain chip at a flow rate of 150 μL/min, and the signal data (brightness, recorded by a CCD camera, indicating the binding affinity) were collected by the manufacturer’s analytical software (Toyobo). The signal intensities of 8 independent data points for each spot were averaged and plotted on the Y-axis.

The interaction between various sugar chains and DENVs was evaluated using a surface plasmon resonance (SPR) imaging system (MultiSPRinter, Toyobo, Osaka, Japan). To select a high-affinity sugar chain for effective capture of DENVs, we used a gold-coated chip containing 48 types of sugar chains immobilized on the surface (see S1 Fig). A culture supernatant of DENV-1, -2, or -4 diluted 20-fold in PBS containing 0.05% Tween 20 (PBS-T) was applied to the chips at a flow rate of 150 μL/min. Virus binding was registered as response units (RUs), and monitored in real time. Fig 1 shows a bar plot of peak heights for the interactions between DENVs with each sugar chain immediately before the change of buffer in the DENV solution to wash buffer. The higher values indicate stronger binding. The DENVs used in this study strongly and specifically bound to chondroitin sulfate type-E (CS-E), heparin (Hep), low molecular weight (LMW) dextran sulfate (DS25), synthetic sulfate partial disaccharides in heparin or chondroitin sulfate. The results are in good agreement with previous reports [26–28]. Based on these data and their common availability, we selected heparin and LMW dextran sulfate for further analysis and immobilization on gold nanoparticles.

### Evaluation of the synthesized SGNPs

**Fig 2:**
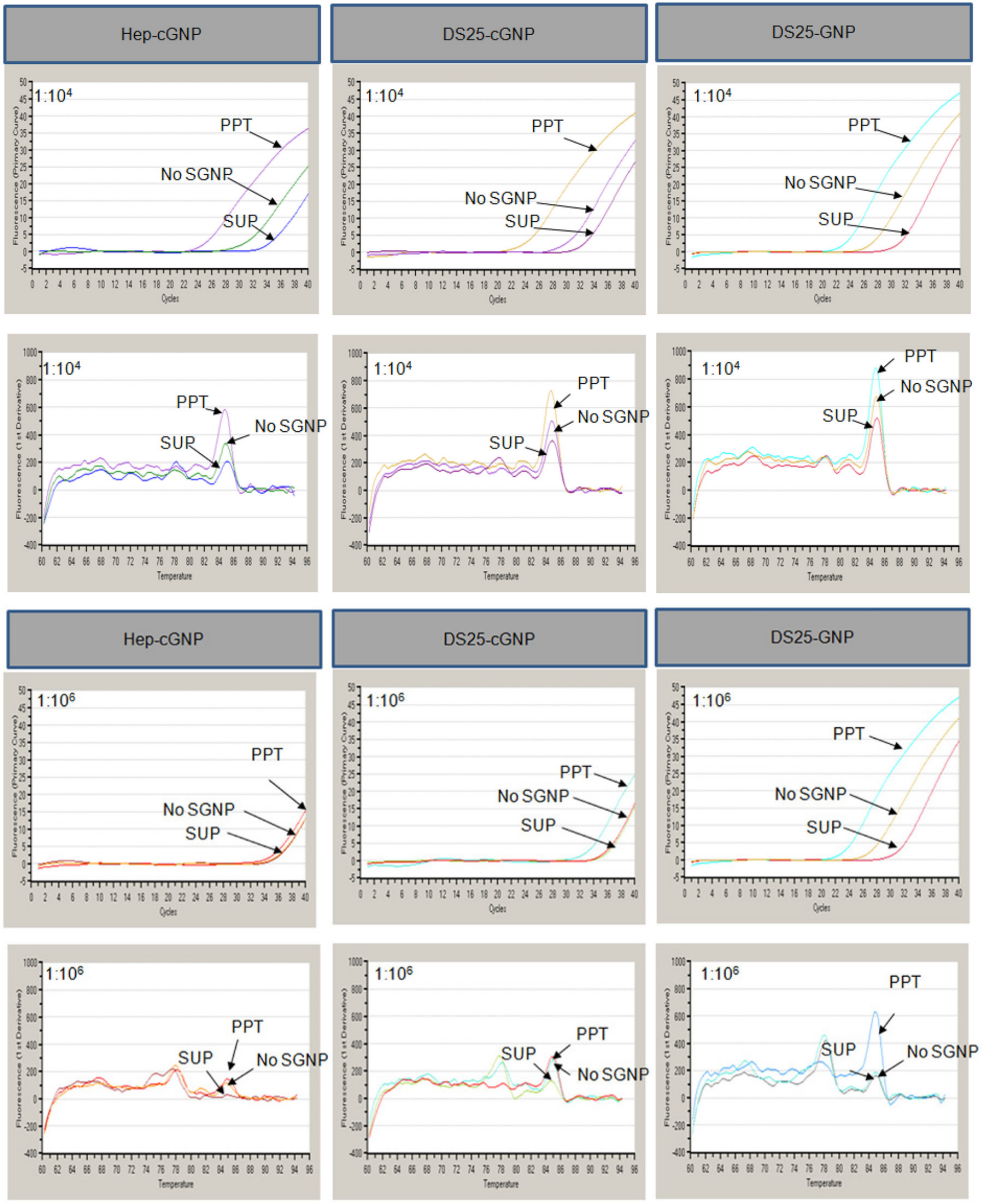
The concentration procedure for dengue virus (DENV) virions using sulfated sugar chain-immobilized gold nanoparticles (SGNPs). A DENV solution was diluted 1:10^4^ or 1:10^6^ in PBS. PPT, precipitate obtained from the treatment with SGNP; SUP, supernatant obtained from the treatment with SGNP; No SGNP, sample without treatment with SGNP. T_m_ for DENV-1 was 85 ± 0.3°C. Upper: fluorescent intensity vs. cycle number (Ct); Lower: melting curve.

Three types of SGNPs (Hep-cGNP, DS25-cGNP, and DS25-GNP) were synthesized as previously reported [24, 25, 29, 30]; we evaluated their ability to capture DENV virions in PBS. Captured DENV virions were quantified by measuring viral RNA by quantitative PCR amplification combined with reverse transcription and SYBR-Green I (RT-qPCR-Syb). Two concentrations of DENV-1 Mochizuki strain (cell culture supernatant diluted 1:10^4^ and 1:10^6^ in PBS) were used as virus input, and the results of the capture are shown in Fig 2 and Table 1. We carefully checked the T_m_ value of DENV-1 to be 84.7–85.1°C. All SGNPs tested captured and concentrated DENV-1 from the sample that had been diluted 1:10^4^. However, at the lower concentration (1:10^6^), we only detected efficient capture of DENV-1 virions with DS25-GNP. Due to the greater sensitivity and efficacy, we used DS25-GNP for further testing.

**Table 1:** Comparison of the SGNPs in terms of their ability to capture and concentrate dengue viruses (DENVs).

SGNP	Dilution	SGNP				No SGNP	
		PPT		SUP			
		Ct	Tm (°C)	Ct	Tm (°C)	Ct	Tm (°C)
Hep-cGNP	x 10^-4^	25.6	84.8	34.9	85.1	31.5	84.9
	x 10^-6^	35.8	78.1	36.8	77.8	36.6	84.8
DS25-cGNP	x 10^-4^	24.5	84.7	33	84.9	30.5	84.8
	x 10^-6^	33.6	85	36.6	84.7	36.2	85
DS25-GNP	x 10^-4^	23.8	84.8	31.6	85	27.6	84.9
	x 10^-6^	29.7	84.9	35.6	78.2	33.7	84.9
Control	PC					22.5	84.8
	NC					35.7	77.7

PPT, precipitate; SUP, supernatant; PC, Positive control: cDNA (plasmid) prepared from the extracted RNA of DENV-1; NC, Negative control: double-distilled water.

### DS25-GNP demonstrates high binding efficiency to DENV in the presence of 1% serum

To evaluate the ability of DS25-GNP to perform under more physiologically relevant conditions, we next tested the ability of DS25-GNP to capture and concentrate DENV virions in the presence of serum. We prepared DENV-2 and -4 suspensions from cell culture supernatants, and diluted the samples 1:10^4^ to 1:10^6^ 1% human serum in PBS. The diluted DENVs were captured and concentrated on DS25-GNP and then quantified using RT-qPCR-Syb. DENVs were detected in the precipitate from all DS25-GNP samples, even at the 1:10^6^ dilution (Table 2; column “PPT”). By contrast, no DENV RNA was detected in “SUP” samples (supernatant after precipitation with SGNP) or “NO SGNP” samples (no treatment with SGNP), except at the higher concentration of DENV (dilution 1:10^4^). These results clearly indicate that DS25-GNP captured and concentrated DENV-2 and -4 in the presence of 1% serum, even at low DENV concentrations.

**Table 2:** Capture and concentration of dengue viruses 2 and 4 (DENV-2 and -4) using DS25-GNP in the presence of 1% human serum.

Virus	Dilution	SGNP				No SGNP	
		PPT		SUP			
		Ct	Tm (°C)	Ct	Tm (°C)	Ct	Tm (°C)
DENV-2	x 10^–4^	31.88	85.42	35.67	85.01	34.04	85.56
	x 10^–5^	34.59	85.08	35.52	78.96	36.53	77.26
	x 10^–6^	36.82	85.15	-	62.19	37.8	78.35
DENV-4	x 10^–4^	31.92	85.39	34.53	84.93	34.04	85.58
	x 10^–5^	34.26	85.11	34.57	78.33	36.5	77.26
	x 10^–6^	36.37	84.93	37.8	78.08	37.6	78.35
Control	PC					22.2	85.08
	NC					33.8	78.47

DENVs were diluted 1:10^4^, 1:10^5^, and 1:10^6^ in PBS. No SGNP means the sample without the treatment with SGNP. PPT, precipitate; SUP, supernatant; PC, Positive control: cDNA (plasmid) prepared from the extracted RNA of DENV-4; NC, Negative control: double-distilled water. Samples positive for DENV were determined by their T_m_ value, i.e., 84.9–85.6°C.

### Evaluation of DS25-GNP from patient samples

#### DENVs can be serotyped on the bases of Tm

**Fig 3:**
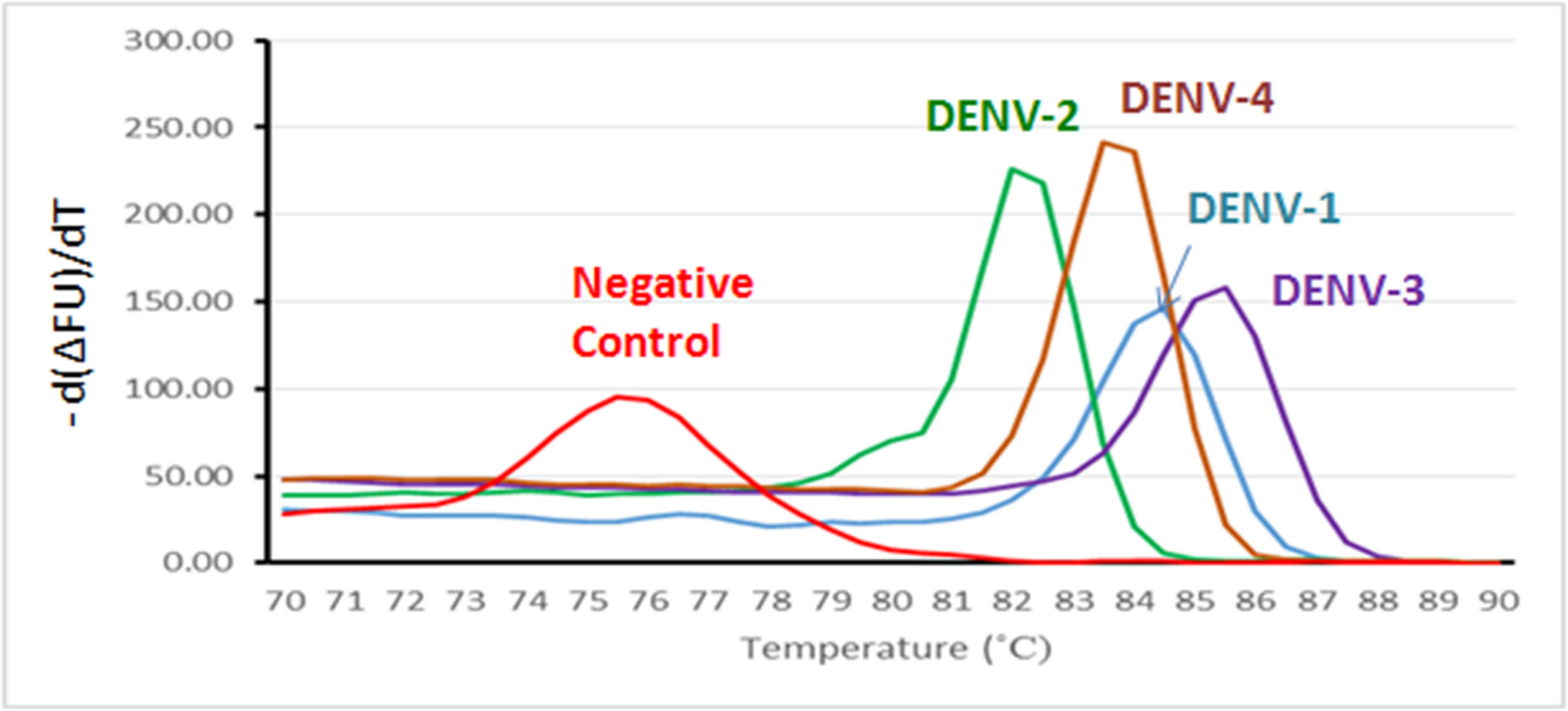
Melting curves for dengue virus (DENV) genotypes 1 through 4 using real-time PCR with SYBR Green I. The peaks of melting curves were clearly separated. T_m_ values for DENV-1, DENV-2, DENV-3, and DENV-4 were 84, 81.5, 85, and 83°C, respectively.

Before we evaluated our detection technique using serum samples from dengue patients, we tested whether our RT-qPCR assay could be performed using protocols previously validated [12]. Purified DENV RNA of each genotype was used as a template for PCR amplification reaction. The peaks of the melting curves of DENV-1, -2, -3, and -4 were 84, 81.5, 85, and 83°C, respectively, whereas the peak of the negative control (NC) curve was 75°C (Fig 3). Poovorawan *et al*. reported that RT-PCR could be used to discriminate between serotypes of DENVs [12]. However, our experiments from Japan using a Takara PCR apparatus found that the specific T_m_ values for all DENVs tested were too similar to one another; therefore, serotyping based on the T_m_ value alone was problematic. On the other hand, our results from experiments performed in Indonesia using a Bio-Rad PCR apparatus, but otherwise under the same conditions, indicate that we were able to determine the DENV serotypes based on their specific T_m_ values. To compare our SGNP method with the gold standard method [9], we tested 22 samples from Pangkal Pinang. Both methods showed the same serotyping results (S1 Table); therefore, we concluded that serotyping with our method was accurate, when using the Bio-Rad PCR machine.

#### Capture and detection of DENVs from sera of patients using SGNPs

The protocol used for clinical research was approved by the Ethics Committee of the Indonesia University, Faculty of Medicine, Department of Microbiology, and the protocol was registered under the number 63/PT02.FK/ETIK/2010. This research was performed according to the Declaration of Helsinki on Human Rights. To determine the efficacy of our new protocols in a clinical setting, we tested serum samples from 87 patients, which were collected from 4 geographic areas in Indonesia: Pangkal Pinang, Lampung, Jakarta, and Solo. Except for the samples from Pangkal Pinang, 65 samples were additionally assayed using IgG-, IgM-, NS1-, and PCR-based methods. All samples were collected in the acute phase of disease (days 1 to 5 after presentation of the first fever).

We compared the efficacy of isolating DENV RNA by SGNP capture with a commercially available kit: the QIAamp Viral RNA Mini Kit from Qiagen (Hilden, Germany). Six microliters of patient serum or plasma was diluted 100 times with PBS, and 500 μL of the diluted solution was mixed with 10 μL of SGNP. The mixture was incubated, and SGNP collected by centrifugation. The precipitate was washed with 500 μL of PBS and resuspended in 20 μL of a 0.1% SDS solution to release RNA from DENV. In parallel, RNA was isolated from 140 μL of the same patient’s serum or plasma using the Qiagen QIAamp kit. RNA from patient samples was extracted and bound to the filter according to the manufacturer’s instructions. Finally, the bound RNA was eluted with 40 μL of the supplied elution buffer. All RNA-containing solutions, which included re-suspended samples of the precipitate, supernatant, diluted serum/plasma without SGNP treatment, and the Qiagen kit-eluted solution, were analyzed by RT-qPCR-Syb.

RNA isolated from the serum and plasma samples of 87 patients using both our SGNP concentration protocol and the Qiagen QIAamp kit were used as templates for our RT-qPCR-Syb protocol. We detected signal for DENV from 25 SGNP samples, and 23 Qiagen kit-eluted samples (Table 3). Among the positive samples, 19 were detected using both the re-suspended SGNP precipitate and the Qiagen kit-eluted solution as input, 6 were detected only using the re-suspended precipitate, and 4 were detected only using the Qiagen kit-eluted solution. Among the 6 samples that tested positive using only the re-suspended precipitate, 4 samples tested negative using other tests, such as the NS1, IgG, and IgM assays. Among the 4 samples that tested positive using only the Qiagen kit-eluted solution, 3 samples also tested positive in the IgM and NS1 assays.

**Table 3:** Positive test results and comparison between the SGNP (sulfated sugar chain-immobilized gold nanoparticles) method and the Qiagen method.

		Qiagen		
		Positive	Negative	Total
SGNP	Positive	19	6	25
	Negative	4	58	62
	Total	23	64	87

Overall, our SGNP method was effective in detecting DENV RNA from only 6 μL of serum or plasma. Using our protocols, we were able to determine that the serotypes of the SGNP-positive samples collected in Pangkal Pinang were DENV-1, -2, and -3. Those from Solo were all DENV-4. It is noteworthy that SGNP-isolated RNA and Qiagen kit-isolated RNA resulted in the detection of different DENV serotypes.

## Discussion

Rapid, early, and highly sensitive methods for DENV detection are being actively developed and improved due to the worldwide prevalence of DENVs, the high risk of DENV infection (such as severe dengue (DHF/DSS)) in some geographic areas, and because of the absence of both a vaccine and an effective drug against DENV [11–15]. We applied our glyco-nanobiotechnology techniques to improve the detection of DENV at very low concentrations in serum (or plasma) samples. This method involves immobilization of DENV on SGNP by capture of the virus with conjugated sugar chains, which concentrates trace amounts of DENV from a clinical sample. This method is based on the well-founded interaction between DENVs and sulfated polysaccharides [19, 21, 22, 23]. To select the sugar chains with the highest affinity for DENVs, we screened 48 types of sugar chains using an array-type Sugar Chip and SPR imaging. We identified heparin and low-molecular-weight dextran sulfate as strong binding partners, and immobilized each of these sugars on gold nanoparticles to prepare the conjugates Hep-cGNP, DS25-cGNP, and DS25-GNP. Of these SGNPs, we identified DS25-GNP as having the best ability to capture and concentrate trace amounts of DENVs. Testing clinical samples demonstrated that our method detects DENV in as little as 6 μL of serum or plasma. Our DS25-GNP method has comparable sensitivity to that of a commercially available RNA extraction method using 140 μL of serum or plasma. As it has been reported that symptoms of severe dengue can be caused in infants by primary infections alone [31, 32], increased surveillance is necessary. As our method requires collecting only trace amount of blood from patients, it can be used to help overcome technical difficulties in screening infants.

After concentration of DENV, we selected the RT-qPCR-Syb method for detection of viral RNA. We chose this method because PCR-based methods have higher sensitivity and accuracy compared with antibody-based methods, such as the assays for NS1, IgG, or IgM. In addition, the use of the intercalator SYBR Green I was expected to work well in a multiplex-like assay for identification of the subtypes of DENV in a single reaction by carefully comparing the T_m_ curves. Our results from clinical testing demonstrated that all subtypes of DENV were detected in Solo, Lampung, and Pangkal Pinang. In a few cases, the subtype of DENV detected in the re-suspended SGNP precipitate was different from that detected in the Qiagen kit-eluted RNA from the same serum sample. The reason for this discrepancy is unclear, but one possible explanation is the difference in the methods used to isolate viral RNA. The re-suspended SGNP precipitate is derived from intact viral particles, whereas the Qiagen kit-eluted RNA represents chemically extracted total RNA/DNA from the serum sample. If this total RNA/DNA contains RNA from destroyed DENV particles previously involved in infection, and which released RNA into plasma/serum, then the above discrepancy may occur. By contrast, our SGNP method provides information related to intact DENV virions, which are the infectious particles. Therefore, the results obtained using the re-suspended SGNP precipitate likely provide information that is more relevant to medical treatment than the Qiagen RNA-extraction method.

Our method is based on the interaction between specific proteins on the surface of the viral particle with low-molecular-weight dextran sulfate on the surface of SGNPs. In the blood of patients with a history of DENV infection, viral particles may interact with DENV-specific antibodies. It was reported that IgM and IgG react with epitopes present on the envelope protein [33, 34, 35]. Our data suggest that IgM prevents the interaction between the virion and SGNP, but not IgG. IgM-positive serum samples from 3 patients found to be DENV-positive using the Qiagen kit-eluted solution were DENV-negative using the re-suspended SGNP precipitate (S2 Table, Lampung No. 9, 13, and 93). However, in an IgG-positive sample, both methods detected DENV (S2 Table, Lampung No 19). Since the numbers of patient samples from our study are limited, further study will be required to determine the relationship between IgM and DENV-SGNP binding.

Most severe cases of dengue occur via primary infection in infants whose mothers have some DENV immunity, and secondary heterotypic infection in children and adults through ADE. According to the ADE model, the viral burden may increase due to the presence of non-neutralizing antibodies. Infection via ADE alters the innate and adaptive intracellular antiviral mechanisms, resulting in unrestricted viral replication and an increase in the serum viral load [32]. This model is supported by studies in mice that are deficient in the ADE-induced type I and II IFN receptors, and which also produce high serum viral loads [5]. Recently, Tithi Pal *et al*. [36] reported the significant correlation between clinical symptoms, IFN-γ, and viral load level. In patients with low levels of IFN-γ, dengue with warning signs correlated with high levels of viral load [36]. Thus, disease severity might be associated with higher viremia levels [37]. However, the viral load in blood is modulated by IgM [38]. Therefore, we predict that in the absence of IgM and with low IFN-γ levels, a high viral load will correlate with the severity of disease, including dengue with warning signs and severe dengue. Our method can detect and serotype small amounts of virus present in the serum, but is inhibited by the presence of IgM. Using our screening method, in combination with additional information such as IgM/IgG and IFN-γ levels in the serum, may provide valuable information that can inform medical treatments. Confirmation of this hypothesis will require *in vivo* studies using mice deficient in type I and II IFN receptors.

## Supporting Information

S1 Fig The maps of sugar chains immobilized on the gold-coated chip (a), the numbers (1–48) refer to sugar chains in S1 Table; an example of the binding of DENV-1 to sugar chains monitored by CCD camera (b). (TIF)

S1 Table The list of sugar chains tested. (PDF)

S2 Table Detailed information on 87 samples analyzed using SGNPs (sulfated sugar chain-immobilized gold nanoparticles) and the Qiagen PCR assay. Yellow cells indicate the corresponding Tm values for each of serotype. Red cells indicate the severe status of patients. Red ovals indicate positive results.
(PDF)

S3 Table Primers used in this study. (PDF)
