# Supplementary figures and images for: A Highly Sensitive Diagnostic System for Detecting Dengue Viruses Using the Interaction between a Sulfated Sugar Chain and a Virion
Source: PLoS One. 2015 May 26;10(5):e0123981. doi: 10.1371/journal.pone.0123981 (PMC4444282; doi:10.1371/journal.pone.0123981)

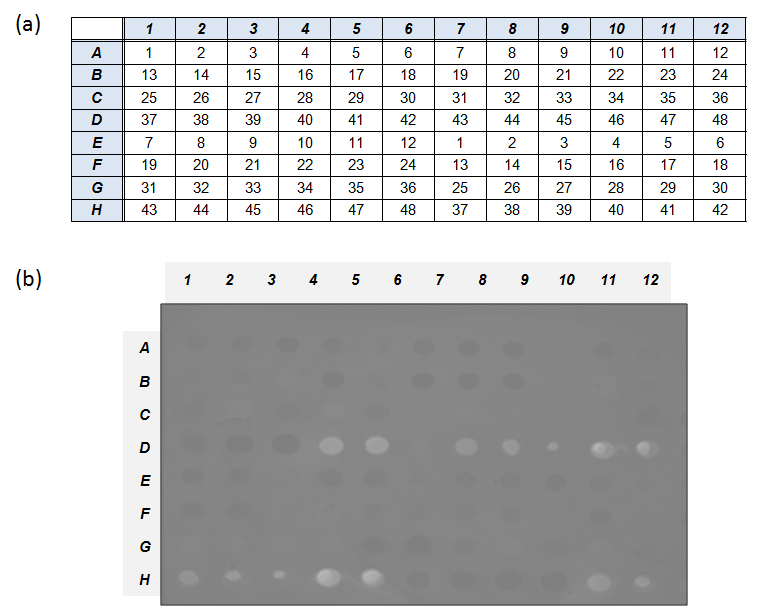

Supplement: S1 Fig — (TIF) [file pone.0123981.s001.tif]
