# Supplementary material for: A Highly Sensitive Diagnostic System for Detecting Dengue Viruses Using the Interaction between a Sulfated Sugar Chain and a Virion
Source: PLoS One. 2015 May 26;10(5):e0123981. doi: 10.1371/journal.pone.0123981 (PMC4444282; doi:10.1371/journal.pone.0123981)
Supplement: S1 Table — (PDF) [file pone.0123981.s002.pdf]

S1 Table. The list of sugar chains tested.

number	sugar chain	number	sugar chain
1	Glcα1-4Glc	25	GalNAcα1-6Glc
2	Glcα1-4Glcα1-4Glc	26	GalNAcβ1-3Gal
3	Glcα1-6Glc	27	NeuAcα2-3Galβ1-4Glc
4	Glcα1-6Glcα1-6Glc	28	NeuAcα2-3Galβ1-4GlcNAc
5	Glcβ1-3Glcβ1-3Glc	29	NeuAcα2-3Galβ1-3GlcNAcβ1-6Glc
6	Glcβ1-4Glc	30	NeuAcα2-3Galβ1-4GlcNAcβ1-6Glc
7	Glcβ1-6Glc	31	NeuAcα2-6Galβ1-4Glc
8	Galα1-6Glc	32	NeuAcα2-6Galβ1-3GlcNAcβ1-6Glc
9	Galα1-4Galβ1-4Glc	33	NeuAcα2-6Galβ1-4GlcNAcβ1-6Glc
10	Galβ1-3GalNAcα1-6Glc	34	NeuAcα2-6GalNAcα1-6Glc
11	Galβ1-4GlcNAcβ1-6Glc	35	Chondroitin
12	Galβ1-4Glc	36	Chondroitin sulfate A
13	Galβ1-4(Fucα1-3)GlcNAcβ1-3Galβ1-4Glc	37	Chondroitin sulfate B
14	Manα1-2Man	38	Chondroitin sulfate C
15	Manα1-3Manα1-6Man	39	Chondroitin sulfate D
16	Manα1-6Man	40	Chondroitin sulfate E
17	Fucα1-2Galβ1-4Glc	41	GlcNS6Sα1-4GlcAβ1-6Glc
18	Fucα1-6Glc	42	GlcNS6Sα1-4IdA2Sα1-6Glc
19	Fucβ1-6Glc	43	GlcNSα1-4GlcAβ1-6Glc
20	Xylβ1-6Glc	44	GlcNSα1-4IdA2Sα1-6Glc
21	GlcNAcα1-6Glc	45	GlcAβ1-3GalNAc4S6Sβ1-6Glc
22	GlcNAcβ1-4GlcNAc	46	Heparin
23	GlcNAcβ1-3Galβ1-4GlcNAcβ1-3Galβ1-4Glc	47	LMW Dextran sulfate
24	GlcNAcβ1-6Glc	48	CM-chitin

Note that ring structures of sugars at reducing-ends were lost during reductive amination to prepare sugar chain ligand-liker conjugate.
