# Supplementary material for: A Highly Sensitive Diagnostic System for Detecting Dengue Viruses Using the Interaction between a Sulfated Sugar Chain and a Virion
Source: PLoS One. 2015 May 26;10(5):e0123981. doi: 10.1371/journal.pone.0123981 (PMC4444282; doi:10.1371/journal.pone.0123981)
Supplement: S2 Table — Yellow cells indicate the corresponding Tm values for each of serotype. Red cells indicate the severe status of patients. Red ovals indicate positive results. (PDF) [file pone.0123981.s003.pdf]

S2 Table. **Detailed information on 87 samples analyzed using SGNPs (sulfated sugar chain-immobilized gold nanoparticles) and the Qiagen PCR assay. Yellow cells indicate the corresponding Tm values for each of serotype. Red cells indicate the severe status of patients. Red ovals indicate positive results.**

City	Sample No	SGNP-PPT			SGNP-SUP			Qiagen			Serotype	NS 1	IgG	IgM	Diagnosis	Gender	Age
		Ct Value	Tm value		Ct Value	Tm value		Ct Value	Tm value								
Pangkal Pinang	1	22.07	85	●	23.39	85	●	18.35	83.5	●	III	No data`s			Fever 3d	M	4
	2	11.73	75	—	31.19	74.5	—	30.49	78.5	—					Fever 1d	F	2
	3	5.03	74.5	—	33.57	75	—	31.29	78	—					Fever 3d	M	11 MONTHS
	4	8.39	81.5	●	27.7	81.5	●	23.33	83.5	●	II				Fever 2d	F	16
	5	26.57	75	—	32.67	74.5	—	31.49	77.5	—					Fever 4d	F	7 MONTHS
	6	31.24	74.5	—	32.05	74.5	—	30.4	79	—					Fever 2d	M	8 MONTHS
	7	33.67	75	—	33.79	75	—	30.55	78	—					Fever 3d	F	2
	8	35.4	75.5	—	35.81	75	—	11.98	84.5	●	I				Fever 2d	M	4
	9	29.1	75	—	30.61	75	—	31.22	77	—					Fever 3d	M	7
	10	15.29	75	—	33.96	75	—	31.74	79.5	—					Fever 3d	F	4
	11	34.73	74.5	—	32.49	74	—	31.06	78	—					Fever 3d	F	12
	12	18.18	81.5	●	31.27	81.5	●	28.22	83.5	●	II				Fever 2d	F	8
	13	25,40	81.5	●	31.38	81	●	27.16	84.5	●	II				Fever 2d	F	20
	14	29.47	74.5	—	34.03	74	—	31.2	79	—					-	M	32
	15	25.77	84.5	●	27.1	84.5	●	31.4	76.5	—	I				-	F	10
	16	35.26	75	—	33.04	74.5	—	31.6	79	—					-	F	1
	17	27.51	84	●	31.01	75	—	25.9	77.5	—	I				-	M	33
	18	27.26	74.5	—	31.12	75	—	28.47	79	—					-	F	20
	19	30.74	75	—	32.05	74	—	30.26	79.5	—					—	M	12
	20	26.63	84.5	●	28.22	85	●	28.34	84.5	●	III				—	M	17
	21	21.85	82	●	23.08	82.5	●	22.37	84.5	●	II				—	M	19
9	22	32.6	84.5	●	31.85	74.5	—	30.62	79	—	I				Fever 3d	M	7
Lampung	19	29.02	81.5	●	29.34	74.5		22.47	81.5	●	II	+	+	—	Fever 3d	F	24
	66	24.28	84.5	●	26.15	84	●	16.95	84.5	●	I	+	—	—	Fever 2d	M	17
	18	23.24	84.5	●	24.69	84.5	●	15.68	85	●	III	+	—	—	Fever 2d	F	1
	37	30.25	75	—	29.78	75	—	29.26	74.5	—		+	—	—	Fever 4d	F	39
	13	30.19	74.5	—	30.15	75	—	27.17	84.5	●	I/III	+	—	+	Fever 3d	M	25
	9	29.88	74.5	—	30.34	74.5	—	28.17	84	●	I	+	—	+	Fever 3d	F	1
	71	19.13	84	●	18.82	84	●	9.27	84	●	I	+	—	—	fever 5d	M	25
	93	31.04	75	—	29.95	75	—	28.88	84	●	I	+	—	+	fever 5d	F	3
	95	29.79	75	—	29.05	74.5	—	28.18	74.5	—		+	+	+	fever 5d	M	35
Jakarta	28	34.04	74.5	—	30.55	74.5	—	33.8	73.5	—		—	—	—	fever 5d	M	17
	29	29.45	74.5	—	30.59	75	—	24.92	74.5	—		—	—	—	—	M	17
	30	31.16	74	—	30.72	74.5	—	28.4	74	—		—	—	+	DHF Gr 2	F	15
	31	29.43	74.5	—	34.13	75	—	0.62	74	—		-	+	-	DSS	F	12
	32	30.99	74.5	—	33.73	75	—	28.9	84	●	I	+	-	-	DHF Gr 1	M	11
	33	30.67	74.5	—	0.67	74.5	—	29.71	74	—		-	-	+	DHF Gr 3	F	7
13	34	30.76	74.5	—	34.06	74.5	—	26.88	74	—		-	-	-	Fever 4d	M	12
Solo	1	34.47	74.5	—	34.52	75	—	29.23	74.5	—		-	-	-	Fever	F	2
	2	30.62	82.5	●	34.65	76	—	25.39	74.5	—	IV	+		-	Fever	M	11
	3	30.62	74.5	—	32.63	75	—	29.97	74.5	—		-	-	-	Fever	M	11
	4	35.05	75	—	33.89	74.5	—	33.5	74.5	—		-	-	-	Fever	M	24
	5	31.57	75	—	33.14	75	—	28.81	74.5	—		-	-	-	Fever 4d	F	8
	6	32.87	74.5	—	33.67	76.5	—	28.57	74.5	—		-	+	+	Fever	M	23
	7	30.98	75	—	32.84	75	—	26.98	74.5	—		-	-	-	Fever	F	23
	8	23.29	84.5	●	31.1	85	●	24.67	84	●	III	+	-	-	Fever	F	28
	9	34.81	75	—	32.14	74.5	—	22.94	74.5	—		+	-	-	Fever	M	7
	10	31.48	83	●	34.82	75	—	28.63	75	—	IV	-	-	-	Fever 1d	M	45
	11	33.58	82	●	27.98	82.5	●	19.94	82	●	II	+	-	-	Fever 1d	M	11
	12	34.35	75	—	33.48	75	—	31.46	74.5	—		-	-	+	Fever	M	20
	13	34.4	74.5	—	33.65	74.5	—	30.01	74.5	—		-	-	-	Fever 2d	M	13
	14	29.59	74.5	—	33.64	75	—	30.34	74.5	—		-	-	-	Fever 3d	F	19
	15	34.18	75	—	33.31	75	—	32.27	72.5	—		-	-	-	Fever	F	62
	16	32.12	74.5	—	34	75	—	33.85	74.5	—		-	-	-	Fever 2d	M	14
	17	31.69	75	—	32.5	75	—	25.6	74.5	—		+	-	-	Fever 2d	M	14
	18	30.92	75.5	—	32.85	74.5	—	30.2	74.5	—		-	-	-	Fever	F	56
	19	31.63	75	—	34.28	75	—	28.12	74.5	—		-	-	-	Fever	F	20
	20	26.98	75	—	32.97	74.5	—	33.42	75.5	—		-	+	-	DHF	F	20
	21	22.5	83	●	28.13	85.5	●	18.31	85	●	IV/III	+	-	-	Fever 1d	M	14
	22	29.39	74.5	—	33.13	75	—	28.67	74.5	—		-	-	-	Fever 2d	F	31
	23	27.05	75	—	33.23	75	—	29.63	74.5	—		-	-	+	DHF	F	9
	24	25.44	74.5	—	35.18	76	—	28.86	74.5	—		-	-	-	Fever	M	67
	25	30.96	75	—	32.81	74.5	—	28.51	74.5	—		-	-	-	Fever	F	21
	26	30.22	75	—	33.69	75	—	28.84	74.5	—		-	-	-	DHF Gr 1	F	2
	27	34.02	74.5	—	32.07	75	—	29.82	74.5	—		-	+	+	DHF	M	8
	28	25.54	82	●	27.66	83	●	18.94	81.5	●	II	+	-	-	Fever	F	3
	29	1.03	83	●	32.25	74.5	—	29.43	74.5	—	IV	-	-	-	Fever 2d	M	14
	30	24.72	75	—	32.92	75.5	—	27.62	75	—		-	+	+	Fever	M	20

	33	31.43	75.5	—	35.28	80.5	—	29.59	74.5	—		-	+	+	DHF	M	14
	34	31	74.5	—	35.16	74.5	—	28.42	74.5	—		-	+	-	Fever	M	43
	35	33.48	75	—	34.9	74.5	—	29.39	75	—		-	+	-	-	F	39
	36	32.11	75	—	34.93	75	—	27.15	74.5	—		-	-	-	Fever	M	30
	37	21.22	83	●	30.63	75	—	30.65	74	—	IV	-	-	-	Fever 3d	F	63
	38	30.7	75	—	30.13	74.5	—	30.26	74	—		-	-	-	Fever	M	17
	39	28.24	83	●	31.63	74.5	—	29.17	74.5	—	IV	-	-	-	Fever	F	16
	40	30.8	75	—	27.76	75.5	—	28.94	74.5	—		+	+	-	DHF	F	11
	41	31.69	75	—	31.09	75.5	—	29.56	74.5	—		-	+	-	Fever	F	15
	42	26.98	84	●	34.04	84	●	27.55	83.5	●	I	+	-	-	Fever	F	10
	43	31.14	75	—	34.87	74.5	—	29.54	74	—		-	-	-	Fever	F	10
	44	30.79	75	—	32.15	75	—	29.88	74.5	—		-	-	-	Fever	F	6
	45	23.17	84	●	28.17	84	●	23.74	83.5	●	I	+	-	-	Fever	M	-
	46	29.21	75	—	25.47	75	—	29.99	74	—		-	-	-	Fever	M	6
	47	30.98	75	—	25.71	74.5	—	28.64	80.5			-	-	+	-	F	-
	48	30.6	75	—	25.63	74.5	—	29.74	75	—		-	+	+	-	M	19
	49	34.06	75	—	25.52	74.5	—	29.61	74.5	—		-	+	+	-	F	33
