# Supplementary material for: A Highly Sensitive Diagnostic System for Detecting Dengue Viruses Using the Interaction between a Sulfated Sugar Chain and a Virion
Source: PLoS One. 2015 May 26;10(5):e0123981. doi: 10.1371/journal.pone.0123981 (PMC4444282; doi:10.1371/journal.pone.0123981)
Supplement: S3 Table — (PDF) [file pone.0123981.s004.pdf]

S3 Table. Primers used in this study.

Serotype Primer	Sequence	Reference
D1	5′-TCAATATGCTGAAACGCGCGAGAAACCG-3′	[9]
D2	5′-TTGCACCAACAGTCAATGTCTTCAGGTTC-3′	
TS1	5′-CGTCTCAGTGATCCGGGGG-3′	
TS2	5′-CGCCACAAGGGCCATGAACAG-3′	
TS3	5′-TAACATCATCATGAGACAGAGC-3′	
TS4	5′-CTCTGTTGTCTTAAACAAGAGA-3′	
**General Primer**	**Sequence**	
Forward	5′-TTAGAGGAGACCCCTCCC-3′	[12]
Reverse	5′-TCTCCTCTAACCTCTAGTCC-3′	
